# Cefepime-Induced Encephalopathy

**DOI:** 10.7759/cureus.13125

**Published:** 2021-02-04

**Authors:** Dinesh Keerty, Naser A Shareef, Asha Ramsakal, Elizabeth Haynes, Misbahuddin Syed

**Affiliations:** 1 Internal and Hospital Medicine, Moffitt Cancer Center, Tampa, USA; 2 Medicine, Lake Erie College of Osteopathic Medicine, Bradenton, USA; 3 Internal Medicine, Moffitt Cancer Center, Tampa, USA; 4 Internal Medicine/ Infectious Disease, Moffitt Cancer Center, Tampa, USA

**Keywords:** encephalopathy, cefepime-induced neurotoxicity

## Abstract

Cefepime, a fourth-generation cephalosporin, remains an essential antibiotic targeting a broad spectrum of Gram-positive and Gram-negative organisms. However, it also remains an important, yet often unrecognized, cause of encephalopathy. We are here to discuss a case of a 74-year-old male with a common bile duct low-grade adenoma who presented to the hospital for lethargy. He was placed on intravenous cefepime for a *Pseudomonas-*infected hepatobiliary abscess. Approximately five days later, the patient’s spouse reported acutely worsening cognitive changes. The cefepime level was significantly elevated at 160 µg/mL. Although not completely understood, cefepime is felt to antagonize gamma-aminobutyric acid A (GABA-A) receptors and possibly inhibit GABA release. This risk is accentuated in patients with underlying renal dysfunction and increased inflammation across the blood-brain barrier. Clinical manifestations include an impaired level of consciousness, delirium, myoclonus, and seizures. The treatment of choice is the cessation of the antibiotic, which resolves the neurotoxicity within approximately 48 hours. It is important to recognize cefepime as a potential culprit of acute-onset encephalopathy in the appropriate clinical setting, and the cessation of therapy would lead to a complete resolution of its associated neurotoxicity.

## Introduction

Due to an increasing shortage of piperacillin/tazobactam, cefepime has been utilized for the empirical treatment of acutely ill infected patients, since it targets a large variety of Gram-positive and Gram-negative bacteria [1]. Particularly in the setting of delayed renal clearance, elevated cefepime levels can lead to encephalopathy, delirium, and seizures [2]. Its ability to cross the blood-brain barrier (BBB) increases the risk of neurotoxicity [3]. We would like to discuss a case of a patient who developed cefepime-induced encephalopathy. 

## Case presentation

**Figure 1:**
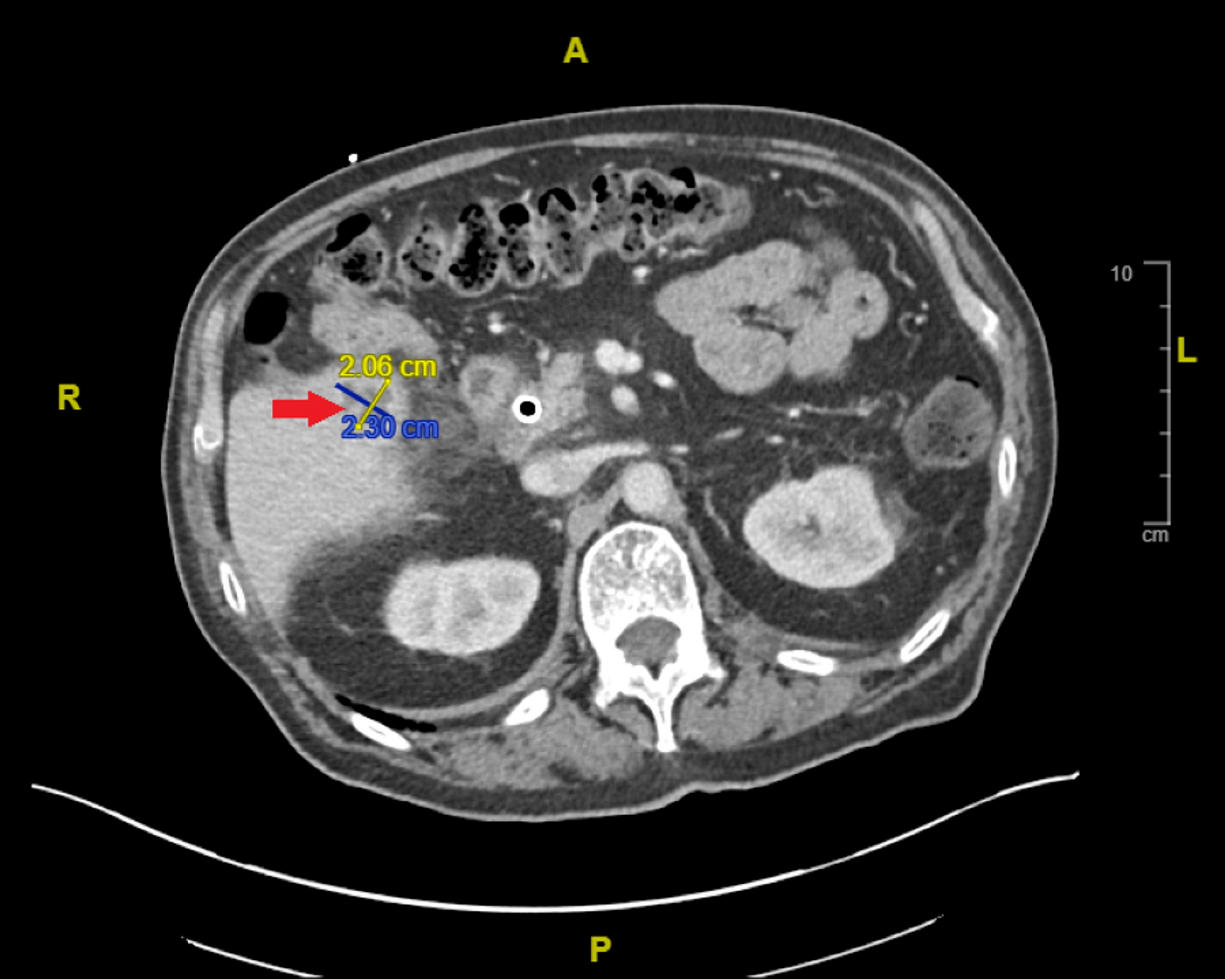
Cross-sectional computed tomography (CT) of abdomen and pelvis with intravenous contrast Red arrow: Findings suggestive of cholangitis with persistent mild right intrahepatic biliary dilation presumably due to stricturing upstream to the common duct stent and new multilocular rim-enhancing segment V lesions concerning for evolving biliary abscesses measuring up to 2.3 cm

A 74-year-old male with a common bile duct low-grade adenoma and chronic kidney disease stage III (estimated glomerular filtration rate 50 mL/min) presented to the hospital for lethargy. Of note, he has some baseline cognitive deficits from a prior lightning strike injury; he can still perform most independent activities of daily living but had to stop working. During his hospitalization, a computed tomography (CT) of the abdomen revealed cholangitis and hepatobiliary abscesses (Figure 1).

Aspirated cultures of these areas grew *Pseudomonas* *aeruginosa* and *Streptococcus* *anginosus*. The minimum inhibitory concentration of cefepime against this *Pseudomonas* species was 8 µg/mL. He was placed on intravenous cefepime, at a dose of 6 g daily via continuous infusion therapy for an anticipated four weeks. He was discharged home to follow up with the infectious disease team.

**Figure 2:**
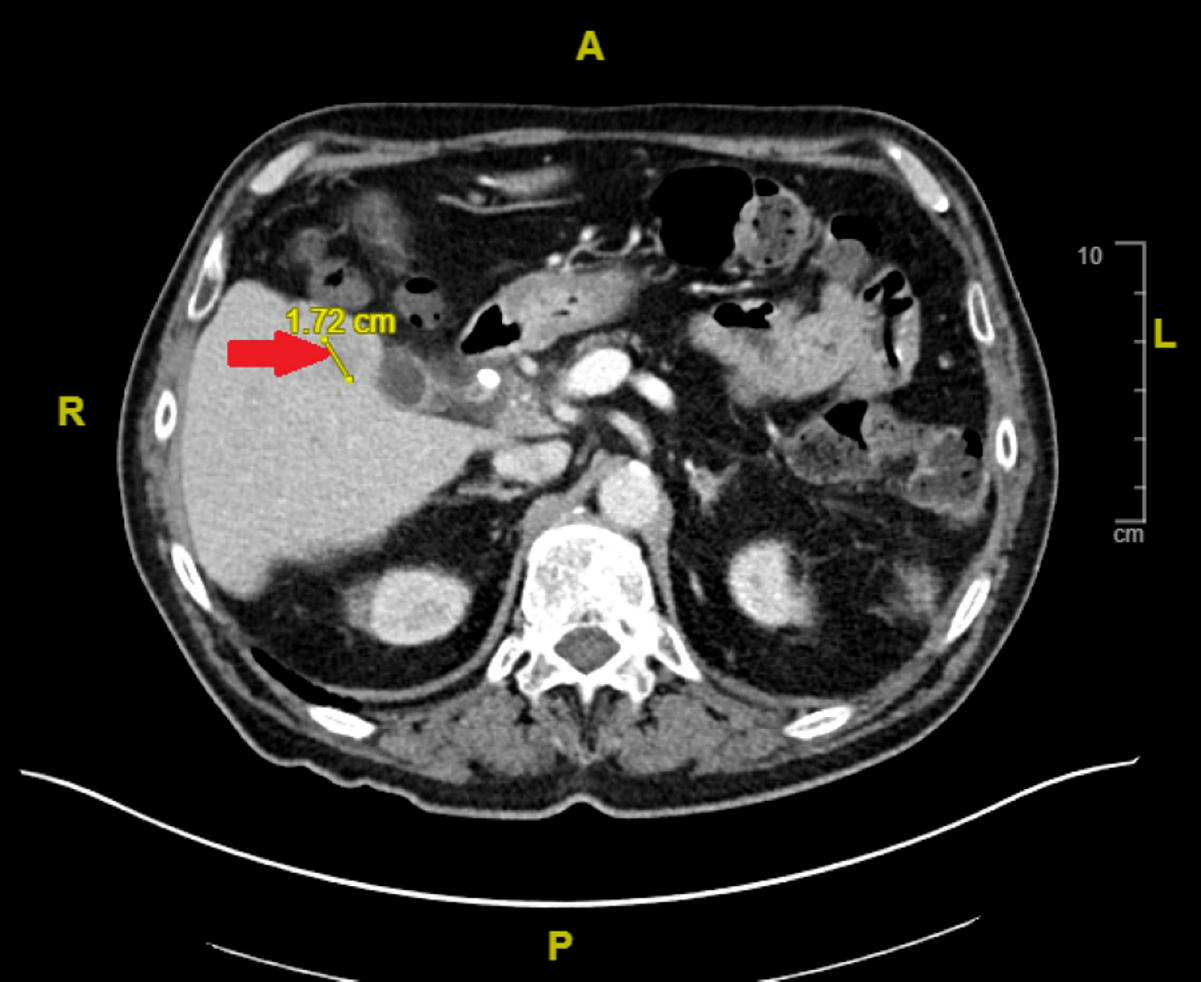
Cross-sectional computed tomography (CT) scan of abdomen and pelvis with intravenous contrast Red arrow: Since the CT from 2.5 weeks ago, there is near resolution of the segment V multilocular lesion adjacent to the gallbladder and interval decrease in the larger inferior segment V multilocular lesion, with no fluid components visualized on the current CT. The slightly low-density residual lesion is approximately 1.8 cm

Five days after discharge, the patient’s spouse reported acutely worsening cognitive changes. He would become lost within his own home, forget how to use equipment such as the television or phone, and make nonsensical comments. He did not have any other motor or sensory deficits. The patient was subsequently re-admitted. Lab and urinalysis values were within normal limits with no obvious signs of infection. CT head imaging revealed no new findings and CT abdominal imaging noted improvement in his hepatobiliary abscesses (Figure 2).

Based on the timeline of events, a concern for cefepime-induced encephalopathy arose, and his antimicrobial was changed to intravenous meropenem. A cefepime level was significantly elevated at 160 µg/mL (normal therapeutic range 5-10 µg/mL). Within two days of changing his antimicrobial regimen, the patient's spouse reported a complete return to his baseline mentation. Patient was discharged to complete 10 days of meropenem treatment.

## Discussion

Cefepime, a fourth-generation cephalosporin, is often used empirically in acutely ill infected patients and targets a large variety of Gram-positive and Gram-negative bacteria. Its neurotoxic potential was first reported in 1999 [2]. Although not entirely elucidated, upon crossing the BBB, the antibiotic may competitively antagonize gamma-aminobutyric acid (GABA) A receptors or inhibit GABA release; this excitatory potential manifests as altered mentation [4]. In normal conditions, approximately 10% of serum cefepime crosses the BBB [5]. But disruptions in the BBB due to inflammation and decreased protein binding capacity can increase central nervous system concentrations of cefepime. Noting that the drug is renally cleared, perhaps the greatest risk of cefepime-induced neurotoxicity is renal impairment coupled with supra-therapeutic dosing; however, 25% of cases have been noted in patients receiving proper doses [5]. Therapeutic serum trough concentrations are felt to be between 5 and 10 µg/mL, but concentrations greater than 20 µg/mL are associated with a significant risk for neurotoxicity [6]. Disproportionately affecting the elderly, cefepime-induced neurotoxicity can occur in up to 15% of critically ill patients, and occurs up to 10 days after initiating therapy with a median of 4-5 days [2]. Symptoms include an impaired level of consciousness, agitation, non-convulsive status epilepticus, seizures, and aphasia [4]. Notably, the appearance of myoclonus in an encephalopathic patient recently placed on cefepime should raise the concern for cefepime neurotoxicity [7]. Treatment involves discontinuing the antibiotic. Anti-epileptic medications may be needed to treat severe cases involving refractory seizures, and hemodialysis may be required to enhance drug clearance. The median time to recovery is within two days [4].

## Conclusions

Clinicians must keep a high index of suspicion for cefepime-induced neurotoxicity in appropriate clinical settings among hospitalized encephalopathic patients, especially in settings of supra-therapeutic dosing, kidney dysfunction, and underlying cognitive deficits.
